# Supplementary material for: Patterns of opioid dose escalation in patients with chronic kidney disease initiated on opioids for the treatment of non-cancer pain
Source: PLoS One. 2026 Mar 20;21(3):e0345309. doi: 10.1371/journal.pone.0345309 (PMC13004407; doi:10.1371/journal.pone.0345309)
Supplement: S1 Fig — (DOCX) [file pone.0345309.s001.docx]

Included follow up period for this study

≥12 months without opioid supply- opioid new user

≥60 days of gap without opioids

Episode 2

Episode 1

|  | | |  | | | | | |  | | |  | | | |  |
| --- | --- | --- | --- | --- | --- | --- | --- | --- | --- | --- | --- | --- | --- | --- | --- | --- |
| Jan | Jun | Dec | Jan | Feb | Mar | Apr |  | Jun |  |  |  | Sep | Oct | Nov | Dec |  |

Start of episode 1

Last day of the day supply

A gap of < 60 days

S1 Fig Illustration of opioid episodes
