# Supplementary material for: Patterns of opioid dose escalation in patients with chronic kidney disease initiated on opioids for the treatment of non-cancer pain
Source: PLoS One. 2026 Mar 20;21(3):e0345309. doi: 10.1371/journal.pone.0345309 (PMC13004407; doi:10.1371/journal.pone.0345309)
Supplement: S1 Table — (DOCX) [file pone.0345309.s002.docx]

# S1 Table ATC codes for opioids and opioid equianalgesic doses

| Opioid analgesics included in the study | |
| --- | --- |
| Buprenorphine | N02AE01 |
| Fentanyl | N02AB03 |
| Ketobemidone | N02AB01 |
| Morphine | N02AA01 |
| Oxycodone | N02AA05 |
| Tapentadol | N02AX06 |
| Tramadol | N02AX02 |
| Opioid analgesics for addiction treatment | |
| Buprenorphine | N07BC01 |
| Methadone | N07BC02 |
| Opioid equianalgesic doses | |
| Buprenorphine patch | 110 |
| Fentanyl patch | 100 |
| Ketobemidone | 1.0 |
| Morphine | 1 |
| Oxycodone | 1.5 |
| Tramadol | 0.1 |
| Tapentadol | 0.4 |

[1][2]

1. Svendsen K, Borchgrevink P, Fredheim O, Hamunen K, Mellbye a., Dale O. Choosing the unit of measurement counts: The use of oral morphine equivalents in studies of opioid consumption is a useful addition to defined daily doses. Palliat Med. 2011;25: 725–732. doi:10.1177/0269216311398300

2. Faculty of Pain Medicine of the Royal College of Anaesthetists. Dose equivalents and changing opioids. 2020 [cited 27 Jan 2025]. Available: https://fpm.ac.uk/opioids-aware-structured-approach-opioid-prescribing/dose-equivalents-and-changing-opioids
