# Supplementary material for: Patterns of opioid dose escalation in patients with chronic kidney disease initiated on opioids for the treatment of non-cancer pain
Source: PLoS One. 2026 Mar 20;21(3):e0345309. doi: 10.1371/journal.pone.0345309 (PMC13004407; doi:10.1371/journal.pone.0345309)
Supplement: S2 Table — (DOCX) [file pone.0345309.s003.docx]

S2 Table ICD-10 codes for comorbidities and cancer diagnoses

| Pain related diagnosis | B02.22, D48.1, F45.4, G43.x, G44.x, G50.x, G54.0, G54.6, , G56.x, G57.7, G58.7, G58.9, G89.0, G89.2, G89.4, G90.5, M00.x-M02.x, M05.x-M08.x, M10.x, M11.x, M12.0, M12.1, M12.5, M12.8, M12.9, M14.x-M19.x, M1A.x, M20.1, M21.61, M21.62, M22.4, M23.x, M24.0, M24.1, M24.20, M25.x, M31.5, M32.x-M36.x, M43.2, M43.8, M45.x, M46.0, M46.1, M46.4, M46.5, M46.8, M46.9, M47.x, M48.0, M48.1-M48.3, M48.8, M48.9, M49.8, M50.x, M51.x, M53.0-M53.3, M53.8, M53.9, M54.03-M54.09, M54.1-M54.6, M54.81, M54.89, M54.9, M60.x-M62.x, M65.x, M67.3, M67.4, M70.0-M70.7, M71.1, M71.3, M71.5, M72.x, M75.x, M76.x, M77.x, M79.0-M79.2, M79.6, M79.7, M79.A, M96.1, R51.X, R52.X, S12.x-S14.x, S22.x-S24.x, S32.x-S34.x, S83.x |
| --- | --- |
| Anxiety disorder | F40-F43 |
| Bipolar disorder | F30, F31 |
| Depressive disorder | F32, F33, F34.1 |
| Opioid use disorder | F11 |
| Other substance use disorder | F13-F16, F18. F19 |
| Schizophrenia spectrum disorder | F20-F25, F28, F29 |
| Alcohol use disorder | F10 |
| Tobacco use disorder | F17, Z72.0 |
| Cannabis use disorder | F12 |

| Cancer diagnoses |  |
| --- | --- |
| Any cancer | C00.x, C01.x, C02.x, C03.x, C04.x, C05.x, C06.x, C07.x, C08.x, C09.x, C10.x, C11.x, C12.x, C13.x, C14.x, C15.x, C16.x, C17.x, C18.x, C19.x, C20.x, C21.x, C22.x, C23.x, C24.x, C25.x, C26.x, C30.x, C31.x, C32.x, C33.x, C34.x, C37.x, C38.x, C39.x, C40.x, C41.x, C43.x, C44.x, C45.x, C46.x, C47.x, C48.x, C49.x, C50.x, C51.x, C52.x, C53.x, C54.x, C55.x, C56.x, C57.x, C58.x, C60.x, C61.x, C62.x, C63.x, C64.x, C65.x, C66.x, C67.x, C68.x, C69.x, C70.x, C71.x, C72.x, C73.x, C74.x, C75.x, C76.x, C77.x, C78.x, C79.x, C80.x, C7A.x, C7B.x, C81.x, C82.x, C83.x, C84.x, C85.x, C86.x, C88.x, C90.x, C91.x, C92.x, C93.x, C94.x, C95.x, C96.x, D00.x, D01.x, D02.x, D03.x, D04.x, D05.x, D06.x, D07.x, D09.x, D10.x, D11.x, D12.x, D13.x, D14.x, D15.x, D16.x, D17.x, D18.x, D19.x, D20.x, D21.x, D22.x, D23.x, D24.x, D25.x, D26.x, D27.x, D28.x, D29.x, D30.x, D31.x, D32.x, D33.x, D34.x, D35.x, D36.x, D37.x, D38.x, D39.x, D40.x, D41.x, D42.x, D43.x, D44.x, D45.x, D46.x, D47.x, D48.x, D3A.x, D49.x |
| Lymphoma | C81.x - C85.x, C88.x, C96.x, C90.0, C90.2 |
| Metastatic cancer | C77.x - C80.x |
| Solid tumour without metastasis | C00.x - C26.x, C30.x - C34.x, C37.x - C41.x, C43.x, C45.x - C58.x, C60.x - C76.x, C97.x |
