# Supplementary material for: Patterns of opioid dose escalation in patients with chronic kidney disease initiated on opioids for the treatment of non-cancer pain
Source: PLoS One. 2026 Mar 20;21(3):e0345309. doi: 10.1371/journal.pone.0345309 (PMC13004407; doi:10.1371/journal.pone.0345309)
Supplement: S3 Table — (DOCX) [file pone.0345309.s004.docx]

S3 Table ATC codes for comedications

| Benzodiazepine or Z hypnotics | ATC: N05B, N05C  N05BA: N05BA01, N05BA02, N05BA04, N05BA05, N05BA06, N05BA09, N05BA12;  N05CD: N05CD02, N05CD03, N05CD05, N05CD08; Clonazepam: N03AE01  N05CF: N05CF01, N05CF02, N05CF03 |
| --- | --- |
| Antidepressants | ATC: N06A  Selective serotonin reuptake inhibitor (N06AB): N06AB03, N06AB04, N06AB05, N06AB06, N06AB08, N06AB10; From other N06A: N06AX16, N06AF03, N06AF04, N06AG02, N06AX02, N06AX03, N06AX06, N06AX11, N06AX12, N06AX18; Other bupropion: N07BA02 |
| Gabapentinoids (gabapentin and pregabalin) | ATC: N03AX12, N03AX16 |
| Antipsychotics | N05AA01 N05AA02, N05AA06, N05AB01,  N05AB02, N05AB03, N05AB04, N05AC01, N05AC02, N05AD01, N05AD03, N05AD05, N05AD08, N05AE03, N05AE04, N05AE05, N05AF01, N05AF03, N05AF05, N05AG02, N05AH01 N05AH02, N05AH03, N05AH04, N05AH05 N05AL01, N05AX08, N05AX12 N05AX13 N05AX15 N05AX16 |
| Nsaids | ATC:M01A |
