# Supplementary material for: Patterns of opioid dose escalation in patients with chronic kidney disease initiated on opioids for the treatment of non-cancer pain
Source: PLoS One. 2026 Mar 20;21(3):e0345309. doi: 10.1371/journal.pone.0345309 (PMC13004407; doi:10.1371/journal.pone.0345309)
Supplement: S4 Table — (DOCX) [file pone.0345309.s005.docx]

S4 Table Adjusted hazard ratio for dose escalation to 50 MME/day( Cox proportional regression)

| eGFR categories | Hazard ratios | *P* | LCI | UCI |
| --- | --- | --- | --- | --- |
| 30≤ eGFR <60 mL/min | 1.075 | 0.125 | 0.980 | 1.180 |
| eGFR <30 mL/min | 1.139 | 0.215 | 0.927 | 1.399 |
| Covariates |  |  |  |  |
| Alcohol use disorder | 1.031 | 0.505 | 0.942 | 1.129 |
| Anxiety disorder | 0.957 | 0.199 | 0.895 | 1.023 |
| Other substance use disorders | 1.279 | 0.000 | 1.139 | 1.437 |
| Pain related conditions | 1.239 | 0.001 | 1.098 | 1.399 |
| Schizophrenia disorder | 1.115 | 0.290 | 0.911 | 1.365 |
| Tobacco use disorder | 1.111 | 0.115 | 0.975 | 1.266 |
| Bipolar disorder | 1.065 | 0.419 | 0.914 | 1.240 |
| Cannabis use disorder | 0.973 | 0.822 | 0.763 | 1.240 |
| Depressive disorder | 0.956 | 0.186 | 0.894 | 1.022 |
| Opioid use disorder | 1.508 | 0.000 | 1.353 | 1.681 |
| Antidepressants | 1.155 | 0.000 | 1.078 | 1.239 |
| Antipsychotics | 0.953 | 0.237 | 0.879 | 1.032 |
| Benzodiazepines | 1.272 | 0.000 | 1.194 | 1.355 |
| Gabapentinoids | 1.540 | 0.000 | 1.451 | 1.633 |
| NSAIDs | 0.914 | 0.013 | 0.852 | 0.982 |
| Age | 0.996 | 0.000 | 0.994 | 0.998 |
| Female gender | 0.831 | 0.000 | 0.786 | 0.879 |

Reference eGFR ≥60mL/min
