# Supplementary material for: Patterns of opioid dose escalation in patients with chronic kidney disease initiated on opioids for the treatment of non-cancer pain
Source: PLoS One. 2026 Mar 20;21(3):e0345309. doi: 10.1371/journal.pone.0345309 (PMC13004407; doi:10.1371/journal.pone.0345309)
Supplement: S5 Table — (DOCX) [file pone.0345309.s006.docx]

S5 Table Adjusted sub hazard ratio for dose escalation to 50 MME/day (competing risk regression)

| eGFR categories | SHR | *P* | LCI | UCI |
| --- | --- | --- | --- | --- |
| 30≤ eGFR <60 mL/min | 0.675 | 0.000 | 0.561 | 0.811 |
| eGFR <30 mL/min | 0.640 | 0.043 | 0.415 | 0.986 |
| Covariates |  |  |  |  |
| Alcohol use disorder | 0.831 | 0.032 | 0.702 | 0.984 |
| Anxiety disorder | 1.224 | 0.002 | 1.079 | 1.389 |
| Other substance use disorders | 0.813 | 0.070 | 0.649 | 1.017 |
| Pain related conditions | 2.469 | 0.000 | 1.858 | 3.282 |
| Schizophrenia disorder | 0.722 | 0.113 | 0.482 | 1.080 |
| Tobacco use disorder | 1.270 | 0.059 | 0.991 | 1.627 |
| Bipolar disorder | 1.473 | 0.003 | 1.139 | 1.904 |
| Cannabis use disorder | 0.776 | 0.279 | 0.491 | 1.227 |
| Depressive disorder | 1.044 | 0.479 | 0.927 | 1.176 |
| Opioid use disorder | 1.583 | 0.000 | 1.298 | 1.930 |
| Antidepressants | 1.216 | 0.005 | 1.062 | 1.392 |
| Antipsychotics | 0.765 | 0.000 | 0.664 | 0.881 |
| Benzodiazepines | 0.886 | 0.037 | 0.790 | 0.992 |
| Gabapentinoids | 2.131 | 0.000 | 1.905 | 2.383 |
| NSAIDs | 2.072 | 0.000 | 1.786 | 2.404 |
| Age | 0.967 | 0.000 | 0.964 | 0.970 |
| Female gender | 0.954 | 0.379 | 0.858 | 1.060 |
