# Supplementary material for: Patterns of opioid dose escalation in patients with chronic kidney disease initiated on opioids for the treatment of non-cancer pain
Source: PLoS One. 2026 Mar 20;21(3):e0345309. doi: 10.1371/journal.pone.0345309 (PMC13004407; doi:10.1371/journal.pone.0345309)
Supplement: S6 Table — (DOCX) [file pone.0345309.s007.docx]

S6 Table Adjusted cumulative incidence of opioid dose escalation to ≥50 MME/day at prespecified time points (Fine–Gray, death as competing event)

|  | eGFR (mL/min) | | | | | |
| --- | --- | --- | --- | --- | --- | --- |
| **Time(year)** | **eGFR ≥60** | **eGFR 30–59** | **eGFR <30** | **eGFR ≥60** | **eGFR 30–59** | **eGFR <30** |
|  | Proportion | | | % | | |
| 0.25 | 0.086 | 0.059 | 0.056 | 8.6 | 5.9 | 5.6 |
| 0.5 | 0.161 | 0.112 | 0.106 | 16.1 | 11.2 | 10.6 |
| 0.75 | 0.199 | 0.139 | 0.132 | 19.9 | 13.9 | 13.2 |
| 1 | 0.222 | 0.156 | 0.148 | 22.2 | 15.6 | 14.8 |
| 2 | 0.257 | 0.182 | 0.173 | 25.7 | 18.2 | 17.3 |
| 3 | 0.270 | 0.191 | 0.183 | 27 | 19.1 | 18.3 |
| 5 | 0.284 | 0.202 | 0.192 | 28.4 | 20.2 | 19.2 |
| 8 | 0.296 | 0.211 | 0.201 | 29.6 | 21.1 | 20.1 |

Predictions from Fine–Gray competing-risks regression (death as competing event); other covariates held at their sample means
