# Supplementary material for: Patterns of opioid dose escalation in patients with chronic kidney disease initiated on opioids for the treatment of non-cancer pain
Source: PLoS One. 2026 Mar 20;21(3):e0345309. doi: 10.1371/journal.pone.0345309 (PMC13004407; doi:10.1371/journal.pone.0345309)
Supplement: S7 Table — (DOCX) [file pone.0345309.s008.docx]

S7 Table Adjusted hazard ratio for dose escalation to 90 MME/day (Cox proportion hazard)

| eGFR categories | Hazard ratios | *P* | LCI | UCI |
| --- | --- | --- | --- | --- |
| 30≤ eGFR <60 mL/min | 1.214 | 0.019 | 1.032 | 1.429 |
| eGFR <30 mL/min | 1.044 | 0.811 | 0.736 | 1.480 |
| Covariates |  |  |  |  |
| Alcohol use disorder | 0.818 | 0.011 | 0.702 | 0.954 |
| Anxiety disorder | 0.976 | 0.676 | 0.873 | 1.092 |
| Other substance use disorders | 1.363 | 0.000 | 1.148 | 1.619 |
| Pain related conditions | 1.275 | 0.03 | 1.024 | 1.589 |
| Schizophrenia disorder | 1.055 | 0.755 | 0.752 | 1.481 |
| Tobacco use disorder | 0.931 | 0.503 | 0.756 | 1.147 |
| Bipolar disorder | 1.095 | 0.454 | 0.864 | 1.388 |
| Cannabis use disorder | 0.952 | 0.787 | 0.665 | 1.363 |
| Depressive disorder | 0.938 | 0.245 | 0.843 | 1.045 |
| Opioid use disorder | 1.566 | 0.000 | 1.347 | 1.821 |
| Antidepressants | 1.376 | 0.000 | 1.209 | 1.566 |
| Antipsychotics | 0.919 | 0.192 | 0.810 | 1.043 |
| Benzodiazepines | 1.514 | 0.000 | 1.360 | 1.684 |
| Gabapentinoids | 1.541 | 0.000 | 1.391 | 1.707 |
| NSAIDs | 0.800 | 0.000 | 0.709 | 0.904 |
| Age | 0.990 | 0.000 | 0.987 | 0.993 |
| Female gender | 0.927 | 0.12 | 0.842 | 1.020 |

Reference eGFR ≥60mL/min
