# Supplementary material for: Patterns of opioid dose escalation in patients with chronic kidney disease initiated on opioids for the treatment of non-cancer pain
Source: PLoS One. 2026 Mar 20;21(3):e0345309. doi: 10.1371/journal.pone.0345309 (PMC13004407; doi:10.1371/journal.pone.0345309)
Supplement: S8 Table — (DOCX) [file pone.0345309.s009.docx]

S8 Table Adjusted sub hazard ratio for dose escalation to 90 MME/day(Competing risk regression)

| eGFR categories | SHR | *P* | LCI | UCI |
| --- | --- | --- | --- | --- |
| 30≤ eGFR <60 mL/min | 0.569 | 0.000 | 0.431 | 0.752 |
| eGFR <30 mL/min | 0.312 | 0.002 | 0.149 | 0.650 |
| Covariates |  |  |  |  |
| Alcohol use disorder | 0.682 | 0.001 | 0.541 | 0.859 |
| Anxiety disorder | 1.403 | 0.000 | 1.180 | 1.669 |
| Other substance use disorders | 0.860 | 0.317 | 0.639 | 1.156 |
| Pain related conditions | 2.664 | 0.000 | 1.792 | 3.961 |
| Schizophrenia disorder | 0.890 | 0.648 | 0.541 | 1.466 |
| Tobacco use disorder | 1.034 | 0.868 | 0.697 | 1.532 |
| Bipolar disorder | 1.227 | 0.221 | 0.884 | 1.704 |
| Cannabis use disorder | 0.527 | 0.034 | 0.291 | 0.953 |
| Depressive disorder | 1.032 | 0.694 | 0.881 | 1.210 |
| Opioid use disorder | 1.966 | 0.000 | 1.548 | 2.497 |
| Antidepressants | 1.383 | 0.002 | 1.129 | 1.694 |
| Antipsychotics | 0.695 | 0.000 | 0.571 | 0.846 |
| Benzodiazepines | 0.979 | 0.790 | 0.836 | 1.146 |
| Gabapentinoids | 2.424 | 0.000 | 2.076 | 2.831 |
| NSAIDs | 2.110 | 0.000 | 1.712 | 2.601 |
| Age | 0.953 | 0.000 | 0.949 | 0.958 |
| Female gender | 1.030 | 0.697 | 0.888 | 1.194 |
