# Supplementary material for: Patterns of opioid dose escalation in patients with chronic kidney disease initiated on opioids for the treatment of non-cancer pain
Source: PLoS One. 2026 Mar 20;21(3):e0345309. doi: 10.1371/journal.pone.0345309 (PMC13004407; doi:10.1371/journal.pone.0345309)
Supplement: S9 Table — (DOCX) [file pone.0345309.s010.docx]

S9 Table Adjusted cumulative incidence of opioid dose escalation to ≥90 MME/day at prespecified time points (Fine–Gray, death as competing event)

|  | eGFR (mL/min) | | | | | |
| --- | --- | --- | --- | --- | --- | --- |
| **Time(year)** | **eGFR ≥60** | **eGFR 30–59** | **eGFR <30** | **eGFR ≥60** | **eGFR 30–59** | **eGFR <30** |
|  | Proportion | | | % | | |
| 0.25 | 0.035 | 0.020 | 0.011 | 3.5 | 2 | 1.1 |
| 0.5 | 0.076 | 0.044 | 0.024 | 7.6 | 4.4 | 2.4 |
| 0.75 | 0.109 | 0.064 | 0.035 | 10.9 | 6.4 | 3.5 |
| 1 | 0.126 | 0.074 | 0.041 | 12.6 | 7.4 | 4.1 |
| 2 | 0.177 | 0.105 | 0.059 | 17.7 | 10.5 | 5.9 |
| 3 | 0.194 | 0.116 | 0.065 | 19.4 | 11.6 | 6.5 |
| 5 | 0.205 | 0.123 | 0.069 | 20.5 | 12.3 | 6.9 |
| 8 | 0.211 | 0.126 | 0.071 | 21.1 | 12.6 | 7.1 |

Predictions from Fine–Gray competing-risks regression (death as competing event); other covariates held at their sample means
