# Supplementary material for: Patterns of opioid dose escalation in patients with chronic kidney disease initiated on opioids for the treatment of non-cancer pain
Source: PLoS One. 2026 Mar 20;21(3):e0345309. doi: 10.1371/journal.pone.0345309 (PMC13004407; doi:10.1371/journal.pone.0345309)
Supplement: Attachment — Submitted filename: PONE-D-25-59036_reviewer.pdf [file pone.0345309.s011.pdf]

PLOS ONE

Patterns of opioid dose escalation in patients with chronic kidney disease initiated on opioids for the treatment of non-cancer pain

--Manuscript Draft--

**Manuscript Number:**	PONE-D-25-59036
**Article Type:**	Research Article
**Full Title:**	Patterns of opioid dose escalation in patients with chronic kidney disease initiated on opioids for the treatment of non-cancer pain
**Short Title:**	Opioid dose escalation in CKD patients
**Corresponding Author:**	Juan Jesus Carrero<br>Karolinska Institutet<br>Stockholm, SWEDEN
**Keywords:**	opioids, dose escalation, chronic kidney disease, pattern
**Abstract:**	Background:<br>Pain management in chronic kidney disease (CKD) is challenging due to altered drug metabolism, impaired excretion, and higher opioid toxicity risk. Despite this, opioids are commonly prescribed, yet real-world data on dose escalation in CKD remain limited.<br>Objective:<br>To investigate patterns and timing of opioid dose escalation to ≥50 and ≥90 MME/day among new opioid users across kidney function levels.<br>Methods:<br>This population-based cohort study used data from the Stockholm Creatinine Measurements (SCREAM) project linking diagnoses, prescriptions, and laboratory records. Adult new opioid users (no prior opioid in 12 months) from 2012–2021 were categorized by baseline eGFR (≥60, 30–59, <30 mL/min/1.73m²). "Opioids were identified using ATC codes, and daily doses (MME/day) were calculated based on strength, quantity, and equianalgesic ratios. Fine–Gray competing-risks regression assessed time to dose escalation (≥50 and ≥90 MME/day), accounting for death as a competing event."<br>Results:<br>Of 81,987 adult new opioid users, 5,987 (7.3%) escalated to ≥50 MME/day comprising 7.4%, 6.8%, and 8.1% of patients with eGFR ≥60, 30–59, and <30 mL/min/1.73m², respectively. For ≥90 MME/day, 2,067 (2.5%) escalated, 2.5%, 2.3%, and 2.9% across the same eGFR categories. Competing risks regression showed significantly lower risks of escalation among patients with reduced eGFR levels. For ≥50 MME/day, the sub distribution hazard ratios (SHRs) were 0.67 (95% CI: 0.56–0.81, p < 0.001) for eGFR 30–59 and 0.64 (95% CI: 0.42–0.99, p= 0.043) for those with eGFR <30.For ≥90 MME/day, SHRs were 0.57 (95% CI: 0.43–0.75, p < 0.001)and 0.31(95% CI: 0.15–0.65, p = 0.002), respectively. Most escalation occurred within six months, with minimal increase thereafter.<br>Conclusion:<br>Opioid dose escalation occurred across all eGFR levels, underscoring the need for cautious, individualized prescribing and close monitoring, especially in patients with reduced kidney function.
**Order of Authors:**	Che Suraya Zin
	Stefania Lando
	Ailema González-Ortiz
	Viyaasan Mahalingasivam
	Shayan Mostafaei
	Wan Rohaidah Ahmad
	Mazlila Meor Ahmad Shah
	Björn Wettermark

Powered by Editorial Manager® and ProduXion Manager® from Aries Systems Corporation

	Juan Jesus Carrero
**Additional Information:**	
**Question**	**Response**
**Financial Disclosure**<br><br>Enter a financial disclosure statement that describes the sources of funding for the work included in this submission. Review the submission guidelines for detailed requirements. View published research articles from *PLOS ONE* for specific examples.<br><br>This statement is required for submission and **will appear in the published article** if the submission is accepted. Please make sure it is accurate.<br><br>**Funded studies**<br>Enter a statement with the following details:<br>• Initials of the authors who received each award<br>• Grant numbers awarded to each author<br>• The full name of each funder<br>• URL of each funder website<br>• Did the sponsors or funders play any role in the study design, data collection and analysis, decision to publish, or preparation of the manuscript?<br><br>Did you receive funding for this work?	The author(s) received no specific funding for this work.
**Competing Interests**<br><br>Use the instructions below to enter a competing interest statement for this submission. On behalf of all authors, disclose any competing interests that could be perceived to bias this work—acknowledging all financial support and any other relevant financial or non-financial competing interests.<br><br>This statement is required for submission and **will appear in the published article** if the submission is accepted. Please make sure it is accurate and that any funding	This research received no specific grant from any funding agency in the public, commercial, or not-for-profit sectors. CSZ was supported by a research grant from The Ministry of Education Malaysia (Fundamental Research Grant Scheme, FRGS/1/2022/SKK16/UIAM/01/3). The funders were not involved in the design of the study and collection, analysis, and interpretation of data and in writing the manuscript

sources listed in your Funding Information later in the submission form are also declared in your Financial Disclosure statement.<br><br>View published research articles from *PLOS ONE* for specific examples.<br><br>**NO authors have competing interests**<br><br>Enter: *The authors have declared that no competing interests exist.*<br><br>**Authors with competing interests**<br><br>Enter competing interest details beginning with this statement:<br><br>*I have read the journal's policy and the authors of this manuscript have the following competing interests: [insert competing interests here]*<br><br>* typeset	
**Ethics Statement**<br><br>Enter an ethics statement for this submission. This statement is required if the study involved:<br><br>• Human participants<br>• Human specimens or tissue<br>• Vertebrate animals or cephalopods<br>• Vertebrate embryos or tissues<br>• Field research<br><br>Write "N/A" if the submission does not require an ethics statement.<br><br>General guidance is provided below. Consult the submission guidelines for detailed instructions. **Make sure that all information entered here is included in the Methods section of the manuscript.**	This study was conducted in accordance with the Declaration of Helsinki and adhered to the STROBE guidelines. Ethical approval for the SCREAM project was originally granted by the Regional Ethics Review Board in Stockholm (reference 2017/793-31), with a subsequent amendment approved by the Swedish Ethical Review Authority (reference 2024-07754-02). The Swedish National Board of Health and Welfare approved the use of national health register data.<br>SCREAM data are pseudo-anonymized before release, and the research team received only fully de-identified datasets. Consequently, informed consent was not required according to Swedish law. The data used in this study were accessed for research purposes on 20 June 2023, and no identifiable information was accessible to the authors during or after data collection.

Powered by Editorial Manager® and ProduXion Manager® from Aries Systems Corporation

**Format for specific study types**<br><br>**Human Subject Research (involving human participants and/or tissue)**<br>• Give the name of the institutional review board or ethics committee that approved the study<br>• Include the approval number and/or a statement indicating approval of this research<br>• Indicate the form of consent obtained (written/oral) or the reason that consent was not obtained (e.g. the data were analyzed anonymously)<br><br>**Animal Research (involving vertebrate animals, embryos or tissues)**<br>• Provide the name of the Institutional Animal Care and Use Committee (IACUC) or other relevant ethics board that reviewed the study protocol, and indicate whether they approved this research or granted a formal waiver of ethical approval<br>• Include an approval number if one was obtained<br>• If the study involved *non-human primates*, add *additional details* about animal welfare and steps taken to ameliorate suffering<br>• If anesthesia, euthanasia, or any kind of animal sacrifice is part of the study, include briefly which substances and/or methods were applied<br><br>**Field Research**<br><br>Include the following details if this study involves the collection of plant, animal, or other materials from a natural setting:<br>• Field permit number<br>• Name of the institution or relevant body that granted permission	
**Data Availability**<br><br>Authors are required to make all data underlying the findings described fully available, without restriction, and from the time of publication. PLOS allows rare exceptions to address legal and ethical concerns. See the PLOS Data Policy and FAQ for detailed information.	No - some restrictions will apply

Powered by Editorial Manager® and ProduXion Manager® from Aries Systems Corporation

A Data Availability Statement describing where the data can be found is required at submission. Your answers to this question constitute the Data Availability Statement and **will be published in the article**, if accepted.<br><br>**Important:** Stating 'data available on request from the author' is not sufficient. If your data are only available upon request, select 'No' for the first question and explain your exceptional situation in the text box.<br><br>Do the authors confirm that all data underlying the findings described in their manuscript are fully available without restriction?	
**Describe where the data may be found in full sentences. If you are copying our sample text, replace any instances of XXX with the appropriate details.**<br><br>• If the data are **held or will be held in a public repository**, include URLs, accession numbers or DOIs. If this information will only be available after acceptance, indicate this by ticking the box below. For example: *All XXX files are available from the XXX database (accession number(s) XXX, XXX.)*.<br>• If the data are all contained **within the manuscript and/or Supporting Information files**, enter the following: *All relevant data are within the manuscript and its Supporting Information files.*<br>• If neither of these applies but you are able to provide **details of access elsewhere**, with or without limitations, please do so. For example:<br><br>*Data cannot be shared publicly because of [XXX]. Data are available from the XXX Institutional Data Access / Ethics Committee (contact via XXX) for researchers who meet the criteria for access to confidential data.*<br><br>*The data underlying the results presented in the study are available from (include the name of the third party*	Data from the Stockholm CREAtinine Measurements (SCREAM) project include sensitive individual-level health information and cannot be publicly shared under EU data protection laws. Access may be granted to qualified researchers upon request and approval from the SCREAM data custodians, subject to ethical review and a data-sharing agreement.

and contact information or URL).<br>• This text is appropriate if the data are owned by a third party and authors do not have permission to share the data.<br><br>* typeset	
Additional data availability information:	

Powered by Editorial Manager® and ProduXion Manager® from Aries Systems Corporation

Manuscript

Click here to access/download;Manuscript;251114_Manuscript_PlosOne.do

Patterns of opioid dose escalation in patients with chronic kidney disease initiated on opioids for the treatment of non-cancer pain

Short title: Opioid dose escalation in CKD patients

Che Suraya Zin [1,2*], Stefania Lando[1], Ailema González-Ortiz[1,3], Viyaasan Mahalingasivam[1,4,5], Shayan Mostafaei[1], Wan Rohaidah Ahmad[6], Mazlila Meor Ahmad Shah[7], Björn Wettermark [8] and Juan-Jesús Carrero[1,9*]

[1]Department of Medical Epidemiology and Biostatistics, Karolinska Institutet, Stockholm, Sweden

[2]Department of Pharmacy Practice, Kulliyyah of Pharmacy, International Islamic University Malaysia, Kuantan, Malaysia

[3] Translational Research Center, Instituto Nacional de Pediatria, Mexico City

[4]Department of Non-Communicable Disease Epidemiology, London School of Hygiene & Tropical Medicine, London, UK

[5]Department of Nephrology & Transplantation, Barts Health NHS Trust, London, UK

[6]Pain management Unit, Department of Anaesthesiology and Intensive Care Hospital Sultanah Nur Zahirah, Kuala Terengganu, Terengganu, Malaysia

[7]Pain management Unit, Department of Anaesthesiology and Intensive Care, Hospital Selayang, Selayang - Kepong Hwy, 68100, Batu Caves, Selangor, Malaysia

[8]Department of Pharmacy, Faculty of Pharmacy, Uppsala University, Box 580, Uppsala 75123, Sweden

[9]Department of Medical Specialist Care, Nephrology Clinic, Danderyd University Hospital, Stockholm, Sweden

*Corresponding author
Email: juan.jesus.carrero@ki.se (JJC)

Email: chesuraya@iium.edu.my (CSZ)

Abstract

Background:

Pain management in chronic kidney disease (CKD) is challenging due to altered drug metabolism, impaired excretion, and higher opioid toxicity risk. Despite this, opioids are commonly prescribed, yet real-world data on dose escalation in CKD remain limited.

Objective:

To investigate patterns and timing of opioid dose escalation to ≥50 and ≥90 MME/day among new opioid users across kidney function levels.

Methods:

This population-based cohort study used data from the Stockholm Creatinine Measurements (SCREAM) project linking diagnoses, prescriptions, and laboratory records. Adult new opioid users (no prior opioid in 12 months) from 2012–2021 were categorized by baseline eGFR (≥60, 30–59, <30 mL/min/1.73m²). "Opioids were identified using ATC codes, and daily doses (MME/day) were calculated based on strength, quantity, and equianalgesic ratios. Fine–Gray competing-risks regression assessed time to dose escalation (≥50 and ≥90 MME/day), accounting for death as a competing event."

Results:

Of 81,987 adult new opioid users, 5,987 (7.3%) escalated to ≥50 MME/day comprising 7.4%, 6.8%, and 8.1% of patients with eGFR ≥60, 30–59, and <30 mL/min/1.73m², respectively. For ≥90 MME/day, 2,067 (2.5%) escalated, 2.5%, 2.3%, and 2.9% across the same eGFR categories. Competing risks regression showed significantly lower risks of escalation among patients with reduced eGFR levels. For ≥50 MME/day, the sub distribution hazard ratios (SHRs) were 0.67 (95% CI: 0.56–0.81, p < 0.001) for eGFR 30–59 and 0.64 (95% CI: 0.42–0.99, $p = 0.043$) for those with eGFR <30.For ≥90 MME/day, SHRs were 0.57 (95% CI: 0.43–0.75, $p < 0.001$)and 0.31(95% CI: 0.15–0.65, $p = 0.002$), respectively. Most escalation occurred within six months, with minimal increase thereafter.

Conclusion:

Opioid dose escalation occurred across all eGFR levels, underscoring the need for cautious, individualized prescribing and close monitoring, especially in patients with reduced kidney function.

Introduction

Patients with chronic kidney disease (CKD) often suffer from chronic pain, affecting up to 60% of patients, particularly in more advanced stages [2]. Managing pain in this population poses unique challenges due to altered pharmacokinetic profiles, heightened drug sensitivity, and the accumulation of active metabolites resulting from impaired renal clearance [3,4]. These physiological differences, along with the burden of polypharmacy and multimorbidity, contribute to an elevated risk of medication-related complications in CKD [5].

Opioid analgesics remain a mainstay in the management of chronic pain in CKD patients, with studies showing that over the course of a year, approximately half of this population receives at least one opioid prescription [4,6]. While opioids may be appropriate for selected cases, inappropriate dosing in people with impaired kidney function can result in serious adverse outcomes, including sedation, respiratory suppression, and fatal overdose [7]. There remains a lack of specific clinical guidelines for safe opioid prescribing in this vulnerable group [8].

To improve opioid safety in the general population, the U.S. Centers for Disease Control and Prevention (CDC) issued guidelines in 2016 discouraging routine dose increases beyond 50 milligram morphine equivalents per day (MME/day) and urging caution above 90 MME/day [7]. Studies in the general population have shown significantly increased overdose risks at these thresholds compared to lower doses [9,10]. While prior research has largely focused on extreme opioid doses (≥100 or ≥200 MME/day) in the general population [9,10], little is known about early escalation to ≥50 and ≥90 MME/day, thresholds that are clinically meaningful and embedded in safety guidelines especially among CKD patients. It is not well studied if these

recommendations are followed in persons with CKD or if overdose risks occur at lower doses in persons with altered drug clearance.

We designed this study using real-world electronic health record data to examine to what extent patients with CKD initiated on opioids get their dosage regimens changed in clinical practice. Our objectives were to describe the patterns of opioid dose escalation to ≥50 MME/day and ≥90 MME/day across eGFR categories and evaluating the timing of dose escalation following the initial opioid prescription among patients stratified by kidney function. We hypothesized that patients with normal kidney function (i.e. estimated glomerular filtration rate [eGFR] ≥60 mL/min/1.73m^2) would be more likely to escalate to higher opioid doses and at a faster rate than those with CKD, reflecting both clinical caution and pharmacologic limitations. Understanding these patterns is essential to support safer prescribing practices and develop more tailored opioid use strategies for patients with CKD.

Materials and Methods

Data source

This observational study utilized data from the Stockholm Creatinine Measurements (SCREAM) project, a population-based database encompassing all individuals in the region of Stockholm, Sweden, who accessed healthcare services between 2012 and 2021 [11]. Laboratory data were linked with several regional and national health registries, including the Stockholm regional healthcare data warehouse (VAL), the Swedish Prescribed Drug Register (PDR), the Cause of Death Register, and the Swedish Renal Register (SRR), enabling comprehensive retrieval of information on demographics, clinical diagnoses, healthcare utilization, and dispensed prescription

drugs. The PDR captures details on all prescriptions across Sweden since July 2005, including drug names (coded using the Anatomical Therapeutic Chemical [ATC] classification), dosage, prescription dates, dispensing dates, and details of the prescribing clinic [12].

Ethics approval

This study was conducted in accordance with the Declaration of Helsinki and adhered to the STROBE guidelines. Ethical approval for the SCREAM project was originally granted by the Regional Ethics Review Board in Stockholm (reference 2017/793-31), with a subsequent amendment approved by the Swedish Ethical Review Authority (reference 2024-07754-02). The Swedish National Board of Health and Welfare approved the use of national health register data.

SCREAM data are pseudo-anonymized before release, and the research team received only fully de-identified datasets. Consequently, informed consent was not required according to Swedish law. The data used in this study were accessed for research purposes on 20 June 2023, and no identifiable information was accessible to the authors during or after data collection.

Study population and study design

This was a cohort study following a new-user design. We identified all patients aged 18 years and above who received at least two opioid prescriptions between January 1, 2012, and December 31, 2021. Patients were required to have not received any opioids in the 12 months preceding their first opioid prescription to qualify as new

opioid users (Figure 1) The first opioid prescription was considered the date of opioid initiation and the study's index date. If a patient had multiple episodes of opioid use during the follow-up period, only their first episode of opioid use was considered (S1 Fig). This study defined an episode as a period of continuous opioid use, beginning with the first prescription and continuing as long as there were no gaps of more than 60 days between consecutive prescriptions. The gap between dispensations was defined as the interval from the end of supply of the previous prescription to the date of the subsequent opioid prescription dispensed. This was considered the first treatment episode. If a gap exceeded 60 days, a new episode was assumed to begin with the next prescription. This approach has also been employed in previous studies.[13,14] We excluded anyone without at least one outpatient measurement of serum/plasma creatinine at the time of opioid therapy initiation or up to 18 months before. Patients were followed until an event, death, disenrollment, or the end of the study (i.e., December 31, 2021), whichever comes first.

We evaluated the following opioids commercially available in Sweden; morphine (ATC: N02AA01), buprenorphine (N02AE01), oxycodone (N02AA05), fentanyl (N02AB03), tramadol (N02AX02), ketobemidone (N02AB01), and tapentadol (N02AX06) (S1 Table). These opioids were identified from The Swedish PDR. Two fixed combination products including codeine/paracetamol and codeine/acetylsalicylic acid, respectively, were excluded because of their low potency, limited escalation potential, variable metabolism, and formulation constraints.

To distinguish prescriptions for buprenorphine and methadone dispensed as medication-assisted treatment (MAT) for opioid use disorder (OUD) from those for analgesia, this study excluded the ATC codes assigned for MAT for buprenorphine (N07BC01) and methadone (N07BC02). Any prescriptions for non-oral and non-

transdermal such as injectable or suppository opioids were also excluded. Since this study included dispensed prescriptions, hospital-administered opioids were not included. No opioids are available without prescription as over the counter (OTC) in Sweden during the time of the study.

Measurement of opioid dose

The period of opioid exposure started on the day the first prescription was dispensed and ended after the end of supply from the last prescription. The number of days that each opioid prescription covers was determined by dividing the quantity supplied by the daily frequency. The total number of days that a patient was covered by opioid medication was calculated by adding up the number of days covered by all of their prescriptions. Any overlapping days of opioid coverage between prescriptions during the study period were subtracted. For multiple opioid prescriptions on the same day for the same patient, only the prescription with the largest day supply was included. All dispensed opioid quantities were included in the total milligrams of morphine equivalent (MME) calculations; overlaps only affected the estimation of days' supply, where stockpiling rules were applied to avoid double counting.[15].

The total morphine equivalents for each prescription during first episode after the index date was calculated by multiplying the quantity of each prescription by the strength in milligrams of opioid per unit dispensed of the prescription. This quantity–strength was then multiplied by equianalgesic ratio of the opioid to derive the MME of the prescription (S1 Table). The individual monthly total opioid dose for each patient was obtained by summing the opioid dose across all prescriptions for a particular patient during the follow-up period after initiating opioid. The mean daily opioid dose in MME

was calculated by dividing the total opioid dose by the total number of days covered with opioid for each patient. Prescriptions with extreme values such as days' supply or doses exceeding two times 99th percentile MME were excluded from the study.

Estimation of glomerular filtration rate (eGFR)

In clinical practice, kidney function is categorised using eGFR [1]. A sustained reduction in eGFR below 60 mL/min/1.73m², is indicative of chronic kidney disease (CKD) [1]. We calculated baseline eGFR using the 2021Chronic Kidney Disease Epidemiology Collaboration (CKD-EPI) equation [16] from the outpatient serum/plasma creatinine value closest to the index opioid prescription date and no longer than 18 months prior. Adjustment for race was not applied. All creatinine assays were calibrated to isotope-dilution mass spectrometry standards, in accordance with manufacturer protocols.

Exposure

The primary exposure was baseline kidney function categorised as no CKD or CKD stages G1/G2 (mild CKD) (eGFR ≥60 mL/min/1.73m²), CKD stage G3 (moderate CKD) (eGFR 30–59 mL/min/1.73m²), and CKD stages G4/G5 (advanced CKD) (<30 mL/min/1.73m²) [1].

Outcomes

The primary outcome was opioid dose escalation to ≥50 MME/day. This included the number of patients who experienced escalation and the time to escalation from initial prescription. A secondary outcome assessed escalation to ≥90 MME/day, a threshold

linked to heightened risk of opioid-related adverse events [7]. To ensure outcomes reflected initial escalation rather than repeated episodes, only the first event of dose escalation was considered for each patient.

Covariates

Information on age, and sex were extracted at the index date. Comorbidities were defined through issued diagnoses (S2 Table) recorded in inpatient, outpatient specialist or primary care. We included only comorbidities with direct clinical relevance to opioid prescribing and dose escalation risk. These included chronic or acute pain, history of use disorders (alcohol use disorder, other substance use disorder, cannabis use disorder, and tobacco use disorder), and mental health disorders (anxiety disorder, bipolar disorder, depressive disorder, and schizophrenia disorder). These comorbidities were identified within one year prior to the index date, a timeframe chosen to ensure temporal relevance to opioid use, which is typically recommended for short-term use. Furthermore, in Sweden, prescriptions are valid for a maximum of one year, and patients with pain or other chronic conditions relevant to this study typically have at least annual follow-up, which supports the choice of a 1-year look-back window.

Comedications were measured as dispensed prescriptions within 6 months before the index date. The following comedications were included in this study; benzodiazepines and Z-hypnotics, non-steroidal anti-inflammatory drugs (NSAIDs), antidepressants, antipsychotics and NSAIDs (S3 Table).

Statistical analysis

Patient characteristics were summarized within each eGFR category using descriptive statistics. Continuous variables were reported as median (interquartile range, IQR) , and categorical variables were expressed as counts and percentages. To assess time to opioid dose escalation at thresholds of ≥50 MME/day and ≥90 MME/day, we first employed Kaplan–Meier curves to graphically depict the probability of remaining below the respective dose threshold over time. Log-rank tests were used to evaluate differences in time to escalation between eGFR categories. Next, we used multivariable Cox proportional hazards regression to estimate cause-specific hazard ratios (HRs) for time to dose escalation, using eGFR ≥60 mL/min as the referent category. The models were adjusted for all identified confounders (see section of covariates). Interaction terms were also tested (e.g., between psychiatric conditions and their treatments, and between sex and age) to assess potential effect modification. The proportional hazards assumption was tested by using Schoenfeld residuals, including assessments of time-dependency. Model fit was further evaluated using Martingale and Cox–Snell residuals.

As death represents a frequent competing event in this population, Kaplan–Meier and Cox models may overestimate the cumulative risk of dose escalation. Therefore, these analyses were used primarily for unadjusted descriptive purposes, while the Fine–Gray competing-risks regression was applied as the main analytical approach to provide more accurate risk estimates.

We also employed Fine–Gray competing-risks regression to model time to opioid dose escalation to ≥50 MME/day and ≥90 MME/day, treating death as the competing event

and reporting sub distribution hazard ratios (SHRs) with 95% CIs. Standard errors were cluster-robust at the patient level. Model-based cumulative incidence functions (CIFs) by eGFR category were obtained from the Fine–Gray model (other covariates held at their sample means) and summarized at prespecified time points (3, 6, 9, 12 months; 2, 3, 5, 8 years). As an unadjusted check, we also estimated Aalen–Johansen CIFs by eGFR and performed a Gray-equivalent global test using an unadjusted Fine–Gray model

To test the stability of the findings, a sensitivity analysis was performed by excluding patients with eGFR values above 105 mL/min/1.73 m^2. These elevated eGFR values could represent hyperfiltration cases or, inaccurate GFR estimation in individuals with low muscle mass, multimorbidity or acute illness. All analyses were conducted using Stata version 19 [17]. Statistical tests were two-sided, and a p-value below 0.05 was considered statistically significant.

Results

Patient Characteristics

A total of 219,480 opioid prescriptions were dispensed to 81,987 unique patients during the study period (Fig 1). Of these patients, 70,199 (85.6%) had eGFR ≥60 mL/min/1.73m², 10,395 (12.7%) had eGFR 30–59 mL/min/1.73m², and 1,393 (1.7%) had eGFR <30 mL/min/1.73m² (Table1). Median age was higher among patients with lower eGFR categories. The proportion of female patients increased with lower eGFR,

from 55.8% in the eGFR ≥60 group to 68.1% and 58.7% in the eGFR 30–59 and eGFR <30 groups, respectively.

Fig 1 Study cohort flowchart

Table 1 Patient demographics

Descriptions	Overall	eGFR ≥60 mL/min/1.73m^2	30≤ eGFR <60 mL/min/1.73m^2	eGFR <30 mL/min/1.73m^2
Total number of patients, n (%)	81987	70199(85.6)	10395(12.7)	1393(1.7)
Age, years, median (IQR)	63(48, 77)	59 (45, 72)	83 (75, 89)	83 (71, 90)
Age group, n (%)				
<=40 years	13,828 (16.9%)	13716 (19.5%)	61 (0.6%)	51 (3.7%)
41-50 years	9,844 (12.0 %)	9679 (13.8%)	116 (1.1%)	49 (3.5%)
51-60 years	13,745 (16.8%)	13291 (18.9%)	366 (3.5%)	88 (6.3%)
61-80 years	28,958 (35.3%)	24909 (35.5%)	3639 (35.0%)	410 (29.4%)
>80 years	15,612 (19.0%)	8604 (12.3%)	6213 (59.8%)	795 (57.1%)
Sex, n (%)				
Male	34,936 (42.6%)	31048 (44.2%)	3312 (31.9%)	576 (41.3%)
Female	47,051 (57.4%)	39151 (55.8%)	7083 (68.1%)	817 (58.7%)
Laboratory values				
Baseline eGFR, median (IQR)mL/min/1.73m2	87.9 (71.4, 101.7)	91 (79, 104)	49 (42, 55)	23 (16, 27)
Diagnoses n (%)				
Chronic or acute pain diagnosis	31,289 (45.8%)	27501 (44.6%)	3347 (56.1%)	441 (56.6%)
Alcohol use disorder	3,696(5.40%)	3484 (5.7%)	190 (3.2%)	22 (2.8%)
Opioid use disorder	1,259(1.84%)	1190 (1.9%)	53 (0.9%)	16 (2.1%)
Other substance use disorder	2,016(2.95%)	1948 (3.2%)	56 (0.9%)	12 (1.5%)
Cannabis use disorder	399(0.58%)	396 (0.6%)	1 (<1%)	2 (0.3%)
Tobacco use	1,461(2.14%)	1347 (2.2%)	98 (1.6%)	16 (2.1%)

Descriptions	Overall	eGFR ≥60 mL/min/1.73m^2	30≤ eGFR <60 mL/min/1.73m^2	eGFR <30 mL/min/1.73m^2
Anxiety disorder	15,448(22.6%)	14216 (23.1%)	1098 (18.4%)	134 (17.2%)
Bipolar disorder	954(1.39%)	915 (1.5%)	29 (0.5%)	10 (1.3%)
Depressive disorder	11,283(16.5%)	10147 (16.5%)	1020 (17.1%)	116 (14.9%)
Schizophrenia spectrum disorder	585(0.86%)	498 (0.8%)	77 (1.3%)	10 (1.3%)
Comedications, n (%)				
Anti-depressants	16983(20.7%)	14402 (20.5%)	2290 (22.0%)	291 (20.9%)
Anti-psychotic	6075 (7.4%)	4919 (7.0%)	1027 (9.9%)	129 (9.3%)
Benzodiazepines or Z hypnotics	12701(15.5%0	10173 (14.5%)	2241 (21.6%)	287 (20.6%)
Gabapentinoids	13108(16.0%)	11574 (16.5%)	1380 (13.3%)	154 (11.1%)
NSAIDs	18917(23.1%)	17541 (25.0%)	1286 (12.4%)	90 (6.5%)

Pain-related diagnoses were common in all eGFR categories, reported in 44.6% of patients with eGFR ≥60, 56.1% in those with eGFR 30–59, and 56.6% in those with eGFR <30. The prevalence of anxiety disorders was lower across lower eGFR, from 23.1% in the eGFR ≥60 group, to 17.2% in the eGFR <30 group. The prevalence of depressive disorders was similar across the three groups, reported in 16.5%, 17.1%, and 14.9%, respectively. Use antidepressant medications was similar across the three categories. However, the use of antipsychotics, and benzodiazepines or Z-hypnotics was higher with lower eGFR. In contrast, the use of NSAIDs and gabapentinoids was highest among patients with eGFR ≥60 and lowest among those with eGFR <30 ml/min/1.73m^2.

Opioid dose escalation to 50 MME/day

A total of 5,987(7.3%) patients experienced opioid dose escalation to ≥50 MME/day during a median follow-up of 14 months (IQR 12.5-37.3). This included 5170 (7.4%) patients in the eGFR ≥60 ml/min/1.73m^2 group, 704 (6.8%) in the eGFR 30–59 group, and 113 (8.1%) in the eGFR <30 group. KM curves showed no between-group difference in time to escalation ≥50 MME/day (log-rank χ^2=2.28, p=0.32) and adjusted Cox likewise found no association with eGFR (S4 Table).

As no significant differences were observed using Kaplan–Meier or Cox models, subsequent analyses focused on the Fine–Gray competing-risks regression, which appropriately accounts for death as a competing event.

However, when using a Fine-Gray competing risks regression model that accounted for death as a competing event, kidney function was significantly associated with the likelihood of dose escalation (Fig 2). Compared to patients with eGFR ≥60 mL/min,

those with eGFR 30–59 had a 33% lower sub distribution hazard of escalation (SHR 0.67, 95% CI: 0.56–0.81, *p* < 0.001), while those with eGFR <30 had a 36% lower sub-hazard (SHR 0.64, 95% CI: 0.42–0.99, *p = 0.043*) (S5 Table). Adjusted time-point cumulative incidence was highest for eGFR ≥60 at every horizon: 3/6/9/12 months = 8.6/16.1/19.9/22.2% (≥60 mL/min) vs 5.9/11.2/13.9/15.6% (30–59) vs 5.6/10.6/13.2/14.8% (<30); 2/3/5/8 years = 25.7/27.0/28.4/29.6% (≥60) vs 18.2/19.1/20.2/21.1% (30–59) vs 17.3/18.3/19.2/20.1% (<30) (S6 Table). The unadjusted Aalen–Johansen CIFs differed significantly across eGFR groups (Gray's test: $\chi^2(2)=264.8$, *p<0.001*), consistent with the adjusted Fine–Gray results.

Fig 2 Model-based cumulative incidence (Fine–Gray): escalation to ≥50 MME/day by eGFR category (death as competing event)

In Fine–Gray competing-risks models adjusted for identified confounders, several factors were associated with a higher sub-hazard of opioid dose escalation to ≥50 MME/day: gabapentinoid use (SHR 2.13, 95% CI 1.91–2.38; *p < 0.001*), antidepressant use (SHR 1.22, 95% CI: 1.06–1.39, *p = 0.005*), NSAID use (SHR 2.07, 1.79–2.40; *p < 0.001*), pain-related diagnoses (SHR 2.47, 1.86–3.28; *p < 0.001*), opioid use disorder (SHR 1.58, 1.30–1.93; *p < 0.001*), anxiety disorders (SHR 1.22, 1.08–1.39; *p = 0.002*), and bipolar disorder (SHR 1.47, 1.14–1.90; *p = 0.003*). In contrast, antipsychotic use (SHR 0.76, 0.66–0.88; *p < 0.001*), benzodiazepine use (SHR 0.89, 0.79–0.99; *p = 0.037*), alcohol use disorder (SHR 0.83, 0.70–0.98; *p = 0.032*), and older age (per-year SHR 0.97, 0.964–0.970; *p < 0.001*) were associated with lower sub-hazards; female gender was not significant (SHR 0.95, 0.86–1.06; *p = 0.38*). No significant associations were also observed for other confounders such as schizophrenia and cannabis use disorder (*p ≥ 0.05*) (S5 Table)

Opioid dose escalation to 90 MME/day

A total of 2,067(2.5%) patients experienced opioid dose escalation to ≥90 MME/day during a median follow-up of 14 months (IQR 10-28). This included 1,785 (2.5%) patients with eGFR ≥60 ml/min, 241 (2.3%) patients with eGFR 30–59, and 41(2.9%) patients with eGFR <30 .KM curves showed no between-group difference for escalation ≥90 MME/day (log-rank χ^2=0.31, p=0.854). In adjusted Cox, eGFR 30–59 mL/min had a modestly higher hazard (HR 1.21, 95% CI 1.03–1.43, *p* = 0.019), while eGFR <30 was not significant (S7 Table).

As no significant differences were observed using Kaplan–Meier or Cox models, subsequent analyses focused on the Fine–Gray competing-risks regression, which appropriately accounts for death as a competing event.

In Fine–Gray competing risks regression accounting for death, both groups with reduced eGFR levels had significantly lower sub distribution hazards of escalation ≥90 MME/day compared to patients with eGFR ≥60 mL/min: patients with eGFR 30–59 had a 43% lower risk (SHR = 0.57, 95% CI: 0.43–0.75*, p < 0.001*), and those with eGFR <30 had a 69% lower risk (SHR = 0.31, 95% CI: 0.15–0.65, *p = 0.002*) (Fig 3) (S8 Table). Adjusted time-point CIFs were highest for eGFR ≥60, intermediate for 30–59, and lowest for <30 at every horizon: 3/6/9/12 months = 3.5/7.6/10.9/12.6% (≥60) vs 2.0/4.4/6.4/7.4% (30–59) vs 1.1/2.4/3.5/4.1% (<30). At 2/3/5/8 years, risks were 17.7/19.4/20.5/21.1% (≥60) vs 10.5/11.6/12.3/12.6% (30–59) vs 5.9/6.5/6.9/7.1% (<30) (S9 Table). The unadjusted Aalen–Johansen CIFs for ≥90 MME/day differed significantly across eGFR groups (Gray's test: $\chi^2(2)$=192.5, p<0.001), consistent with the adjusted Fine–Gray results.

Figure 3 Model-based cumulative incidence (Fine–Gray): escalation to ≥90 MME/day by eGFR category (death as competing event)

Concerning identified confounders, adjusted Fine–Gray competing-risks models showed that several covariates were associated with a higher sub distribution hazard of dose escalation ≥90 MME/day: pain-related diagnoses (SHR 2.66, 95% CI 1.79–3.97; $p < 0.001$), opioid use disorder (1.97, 1.55–2.50; $p < 0.001$), anxiety disorders (1.40, 1.18–1.67; $p < 0.001$), antidepressant use (1.38, 1.13–1.69; $p = 0.002$), gabapentinoid use (2.42, 2.08–2.83; $p < 0.001$), and NSAID use (2.11, 1.71–2.60; $p < 0.001$). In contrast, antipsychotic use (0.70, 0.57–0.85; $p < 0.001$), alcohol use disorder (0.68, 0.54–0.86; $p = 0.001$), cannabis use disorder (0.53, 0.29–0.95; $p = 0.034$), and older age (0.95, 95% CI 0.949–0.958; $p < 0.001$)(S8 Table) were associated with lower sub distribution hazards. Other confounders such as schizophrenia and substance-use, benzodiazepine use, and female sex were not statistically significant.

Sensitivity Analyses

In sensitivity analyses, exclusion of patients with eGFR >105 mL/min/1.73 m² did not materially alter the findings, indicating that the main results were robust to potential misclassification in this subgroup.

Discussion

This large population-based study provides insights into patterns of opioid dose escalation in clinical practice by varying degrees of kidney function. We found that patients with CKD had lower risks of opioid dose escalation across both thresholds

(≥50 and ≥90 MME/day). The pattern was consistent at all prespecified time points, with escalation concentrated in the first year particularly by 6 months and only modest accrual thereafter.

In competing risks analysis, opioid dose escalation was less likely in patients with reduced kidney function (eGFR 30-59 and eGFR <30mL/min). These findings align with prior literature emphasizing cautious opioid use in CKD. Conservative opioid prescribing in CKD is recommended due to altered drug clearance and increased toxicity risk [5,8]. Prior work has documented high variability in opioid use among CKD patients thatoften attributed to prescriber hesitation [4] and lower opioid dosing and reduced long-term use in patients with CKD, consistent with our observation of less frequent dose escalation in this population.

The timing of opioid dose escalation in the current study highlights a critical early window in which upward titration occurs. At 6 months, the adjusted cumulative incidence of escalation was 16.1%/11.2%/10.6% for ≥50 MME/day and 7.6%/4.4%/2.4% for ≥90 MME/day in the eGFR ≥60/30–59/<30 mL/min groups, respectively. A study in chronic non-cancer pain patients observed escalation to the much higher threshold of ≥200 MME/day occurring over a median of 186 days (approximately 6.1 months) [18]. This suggests that escalation to ≥50 or ≥90 MME/day in our cohort occurred over comparable or even longer periods, despite representing significantly lower dose thresholds. The slightly longer escalation times in our cohort are expected, as patients with impaired kidney function typically require slower titration due to their increased sensitivity to opioids and elevated risk of adverse drug effects. These findings underscore the importance of early monitoring, especially in the first 4–8 months after opioid initiation, when the risk of dose escalation appears to be highest. This early period represents a crucial opportunity for clinicians to reassess

pain control strategies and reinforce safer prescribing practices, particularly in populations at elevated risk of harm.

Despite clinical caution, high-risk opioid dose escalation still occurred in this cohort. While direct comparisons with previous studies are limited due to differences in population and dose thresholds, most existing literature focuses on extreme escalation, such as ≥100 or ≥200 MME/day. For instance, a study in patients with chronic non-cancer pain reported that 589 patients (1.8%) escalated to ≥200 MME/day [18], while Zin et al. (2019) found that 248 patients (5.3%) reached ≥100 MME/day and 69 (1.5%) exceeded ≥200 MME/day[19]. The lower proportion of patients in the current study reaching ≥90 MME/day (2.5%) likely reflects a more cautious prescribing approach in CKD

These escalation thresholds are clinically important, given the well-documented dose-dependent risks associated with opioids. Bohnert et al. (2011) found that patients prescribed 50–99 MME/day had a 3.7-fold increased risk of opioid overdose death, rising to 8.9-fold at ≥100 MME/day [10]. Similarly, Gomes et al. (2011) reported a 1.9-fold increased risk at 50–99 MME/day and a 2.04-fold increase at ≥100 MME/day [9]. These findings reinforce the rationale behind the CDC guidelines, which discourage exceeding 50 MME/day and recommend extreme caution beyond 90 MME/day especially in high-risk populations such as those with impaired kidney function [7].

Although the Swedish national treatment recommends that opioid prescribing should be individualized and guided by a comprehensive patient assessment rather than fixed numerical dose thresholds [20], the CDC cut-offs of ≥50 and ≥90 MME/day are widely cited in the scientific literature and serve as internationally recognized risk indicators, enabling comparability across studies. These thresholds are also endorsed by the

European Pain Federation (EFIC) as reference points for cautious opioid prescribing [21].

Several covariates were significantly associated with opioid dose escalation, consistent with prior studies. Use of gabapentinoids, NSAIDs, antidepressants, pain-related diagnoses, opioid use disorder and anxiety disorders likely reflects more complex clinical needs, which may predispose patients to higher opioid requirements and greater overdose risk [9,10,22,23]. These findings reinforce the importance of closely monitoring patients with such risk profiles.

We do not interpret the inverse associations (e.g., antipsychotics, benzodiazepines, alcohol/cannabis use, older age) as protective effects. Rather, they reflect selection into more conservative treatment pathways (cautious titration, deprescribing, or alternative therapies) and higher competing mortality, which together reduce the cumulative incidence of escalation in Fine–Gray analyses. Notably, literature linking benzodiazepines to overdose risk addresses a different outcome; our endpoint is dose escalation, so inverse or null associations here are compatible with elevated overdose risk reported elsewhere. Overall, these findings support tailored monitoring in patients with markers of more complex pain management while cautioning against causal interpretation of medication covariates in observational competing-risk analyses.

Strengths and Limitations

One of the key strengths of this study lies in its novel contribution to the evidence base, as it is among the first to comprehensively examine individual patient level data of opioid dose escalation patterns in patients with different eGFR categories. While prior

studies have largely focused on high-dose thresholds (e.g., ≥100 or ≥200 MME/day), no studies to date have assessed dose escalation to ≥50 MME/day and ≥90 MME/day, thresholds that are clinically significant and directly aligned with CDC guidelines. This distinction is particularly important in a CKD population, given the altered drug metabolism, heightened toxicity risk, and unique prescribing challenges in this population. Another key strength of this study is the use of prescribed opioid dose, which reflects actual clinical practice. Unlike defined daily dose (DDD) or package's DDD (FORPDDD), it accounts for the specific dosing interval and duration prescribed, providing a more accurate estimate of opioid exposure.

The study also benefits from a large, population-based cohort, leveraging linked healthcare and prescription registries to capture high-quality real-world data on opioid prescribing across a full spectrum of kidney function. The long follow-up period and complete prescription records enabled precise estimation of opioid dosing trajectories and robust adjustment for a wide array of covariates, including comorbidities and co-medications. Additionally, the application of competing risks regression accounting for death as a competing event adds methodological rigor, particularly relevant in CKD where mortality risk is elevated.

Nonetheless, several limitations must be considered. First, reliance on prescription data does not confirm actual medication use. Second, data on over-the-counter medications, non-pharmacologic pain interventions, and pain severity were unavailable. Although no opioids are available OTC in Sweden, the lack of data on other analgesics including paracetamol could still influence both prescribing behaviour and dose escalation. Third, pain and mental-health diagnoses are under-recorded in administrative data; despite a 1-year ascertainment window before initiation, some conditions were likely underestimated as noted in prior Swedish registry work on

pregabalin use [24], leaving scope for residual confounding. Fourth, codeine was not captured; patients may switch between codeine and other opioids. In Sweden, codeine is mainly available in fixed-dose combinations with paracetamol or aspirin, so the impact on our escalation estimates is likely limited. Additionally, kidney function was assessed at baseline at the time of opioid initiation. Changes in renal function over time may have influenced opioid dose escalation, which was not captured in this study. Finally, the relatively small number of patients with eGFR <30 mL/min may reduce statistical power and limit the generalizability of findings for those with advanced CKD. Overall, these limitations would tend to dilute rather than create effects. The consistent patterns across both thresholds, the early clustering of risk in the CIFs, and robust sensitivity analyses indicate that our central finding on the lower cumulative incidence of escalation with reduced eGFR when accounting for competing death is unlikely to be materially affected.

Conclusion

Our findings indicate that while patients with reduced kidney function had a significantly lower adjusted risk of escalating to high opioid doses, dose escalation still occurred across all levels of eGFR including among those with eGFR <30 mL/min highlighting the need for cautious and individualized prescribing in this vulnerable population. These results address a critical gap in pain management, where clinical guidelines for opioid use in CKD remain lacking. By highlighting patterns of escalation and identifying key risk factors, our study offers valuable insight to support safer, evidence-informed prescribing decisions. In light of the well-documented risks associated with high-dose opioid use including overdose and mortality, it is imperative

that prescribers select the lowest effective dose, tailored to each patient’s renal function and clinical complexity.

Looking ahead, future research should evaluate whether opioid doses are being adequately adjusted in response to worsening kidney function and whether current prescribing practices align with patients' comorbidity burden. Developing renal-specific opioid prescribing frameworks could greatly enhance the safety and effectiveness of pain management in CKD ultimately improving outcomes for a growing and vulnerable patient population.

References

1. KDIGO 2024 Clinical Practice Guideline for the Evaluation and Management of Chronic Kidney Disease. Kidney Disease: Improving Global Outcomes (KDIGO). 2024. Available: https://kdigo.org/wp-content/uploads/2024/03/KDIGO-2024-CKD-Guideline.pdf
2. Lambourg E, Colvin L, Guthrie G, Murugan K, Lim M, Walker H, et al. The prevalence of pain among patients with chronic kidney disease using systematic review and meta-analysis. Kidney International. 2021. pp. 636–649. doi:10.1016/j.kint.2021.03.041
3. Didik S, Golosova D, Xu B, Staruschenko A. Opioids and the Kidney: A Compendium. Kidney360. 2023;4: 1816–1823. doi:10.34067/KID.0000000000000291
4. Lambourg E, Colvin L, Guthrie G, Walker H, Bell S. Analgesic use and associated adverse events in patients with chronic kidney disease: a systematic review and meta-analysis. Br J Anaesth. 2022;128: 546–561. doi:10.1016/j.bja.2021.08.035
5. Odoma VA, Pitliya A, AlEdani E, Bhangu J, Javed K, Manshahia PK, et al. Opioid Prescription in Patients With Chronic Kidney Disease: A Systematic Review of Comparing Safety and Efficacy of Opioid Use in Chronic Kidney Disease Patients. Cureus. 2023;15: 1–14. doi:10.7759/cureus.45485
6. Coluzzi F, Caputi FF, Billeci D, Pastore AL, Candeletti S, Rocco M, et al. Safe use of opioids in chronic kidney disease and hemodialysis patients: Tips and tricks for non-pain specialists. Ther Clin Risk Manag. 2020;16: 821–837. doi:10.2147/TCRM.S262843
7. Dowell D, Haegerich T, Chou R. CDC Guideline for prescribing opioids for chronic pain—United States. JAMA. 2016;315: 1624–1645.
8. Owsiany TM, Hawley CE, Triantafylidis LK, Paik JM. Opioid Management in Older Adults with Chronic Kidney Disease: A Review. AM J Med. 2019;132: 1386–1393. doi:10.1016/j.amjmed.2019.06.014.Opioid
9. Gomes T, Mamdani MM, Dhalla IA, Michael Paterson J, Juurlink DN. Opioid dose and drug-related mortality in patients with nonmalignant pain. Arch Intern Med. 2011;171: 686–691. doi:10.1001/archinternmed.2011.117
10. Bohnert ASB, Valenstein M, Bair MJ, Ganoczy D, McCarthy JF, Ilgen MA, et al. Association Between Opioid Prescribing Patterns and Opioid Overdose-Related Deaths. JAMA. 2011;35: 1315–1321.
11. Carrero JJ, Elinder CG. The Stockholm CREAtinine Measurements (SCREAM) project: Fostering improvements in chronic kidney disease care. J Intern Med. 2022;291: 254–268. doi:10.1111/joim.13418
12. Wettermark B, Hammar N, MichaelFored C, Leimanis A, Petra Otterblad Olausson UB, Persson I, et al. The new Swedish Prescribed Drug Register—Opportunities for pharmacoepidemiological research and experience from the first six months. Pharmacoepidemiol Drug Saf. 2006;16: 726–35.
13. Schulson LB, Dick A, Sheng F, Stein BD. An Exploratory Analysis of Differential Prescribing of High-Risk Opioids by Insurance Type Among Patients Seen by the Same Clinician. J Gen Intern Med. 2023;38: 1681–1688. doi:10.1007/s11606-023-08025-6

14. Larochelle MR, Liebschutz JM, Zhang F, Ross-Degnan D, Wharam JF. Opioid prescribing after nonfatal overdose and association with repeated overdose: A cohort study. Ann Intern Med. 2016;164: 1–9. doi:10.7326/M15-0038

15. Gimeno GS, Mueller T. Assessment of medication adherence in databases. Drug Utilization Research: Methods and Applications: Second Edition. West Sussex: Wiley Blackwell; 2024. pp. 397–405. doi:10.1002/9781119911685.ch39

16. Levey AS, Schmid LASCH, Zhang Y (Lucy) AFC, Feldman HI, Kusek JW, Eggers P, et al. A new equation to estimate glomerular filtration rate. Ann Intern Med. 2009;150: 604–612.

17. StataCorp. Stata: Release 17. College Station, TX: StataCorp LLC; 2023.

18. Kaplovitch E, Gomes T, Camacho X, Dhalla IA, Mamdani MM, Juurlink DN. Sex differences in dose escalation and overdose death during chronic opioid therapy: A population-based cohort study. PLoS One. 2015;10: 1–11. doi:10.1371/journal.pone.0134550

19. Zin CS, Alias NE, Taufek NH, Ahmad MM. Sex differences in high opioid dose escalation among Malaysian patients with long term opioid therapy. J Pain Res. 2019;12: 1251–1257. doi:10.2147/JPR.S199243

20. Läkemedelsverket. Läkemedelsbehandling vid långvarig smärta hos barn och vuxna – behandlingsrekommendation. Inf från Läkemedelsverket. 2017;28. Available: https://www.lakemedelsverket.se/sv/behandling-och-forskrivning/behandlingsrekommendationer/smarta/lakemedelsbehandling-vid-langvarig-smarta-hos-barn-och-vuxna

21. Häuser W, Morlion B, Vowles KE, Bannister K, Buchser E, Casale R, et al. European* clinical practice recommendations on opioids for chronic noncancer pain – Part 1: Role of opioids in the management of chronic noncancer pain. Eur J Pain. 2021;25: 949–968. doi:10.1002/ejp.1736

22. Henry SG, Wilsey BL, Melnikow JA, Iosif A-M. Dose Escalation during the First Year of Long-Term Opioid Therapy for Chronic Pain. Pain Med. 2015;16: 733–744. doi:10.1177/0022146515594631.Marriage

23. Fugelstad A, Ramstedt M, Thiblin I. Oxycodone-related deaths in Sweden 2006 – 2018. 2022;234.

24. Wettermark B, Brandt L, Kieler H, R Bodén. Pregabalin is increasingly prescribed for neuropathic pain, generalised anxiety disorder and epilepsy but many patients discontinue treatment: an observational study. Int J Clin Pr. 2014;68: 104–110. doi:doi:10.1111/ijcp.12182.

List of supporting information

S1 Table ATC codes for opioids and opioid equianalgesic doses

S2 Table ICD-10 codes for comorbidities and cancer diagnoses

S3 Table ATC codes for comedications

S4 Table Adjusted hazard ratio for dose escalation to 50 MME/day(Cox proportional regression)

S5 Table Adjusted sub hazard ratio for dose escalation to 50 MME/day (competing risk regression)

S6 Table Adjusted cumulative incidence of opioid dose escalation to ≥50 MME/day at prespecified time points (Fine–Gray, death as competing event)

S7 Table Adjusted hazard ratio for dose escalation to 90 MME/day (Cox proportion hazard)

S8 Table Adjusted sub hazard ratio for dose escalation to 90 MME/day(Competing risk regression)

S9 Table Adjusted cumulative incidence of opioid dose escalation to ≥90 MME/day at prespecified time points (Fine–Gray, death as competing event)

Fig 1

Click here to access/download;Figure;Fig 1.docx

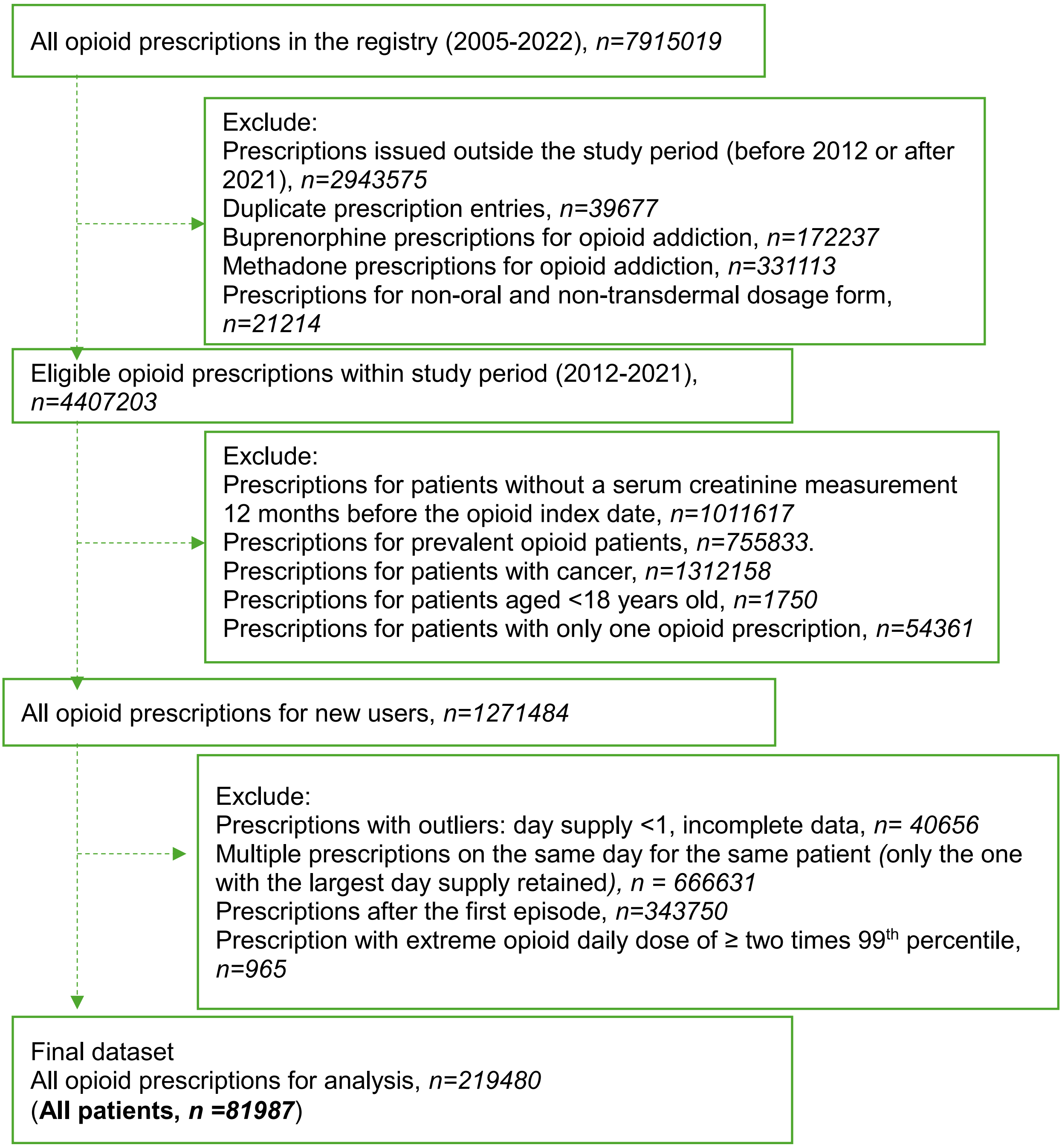

Fig 2

Click here to access/download;Figure;Fig 2.jpg

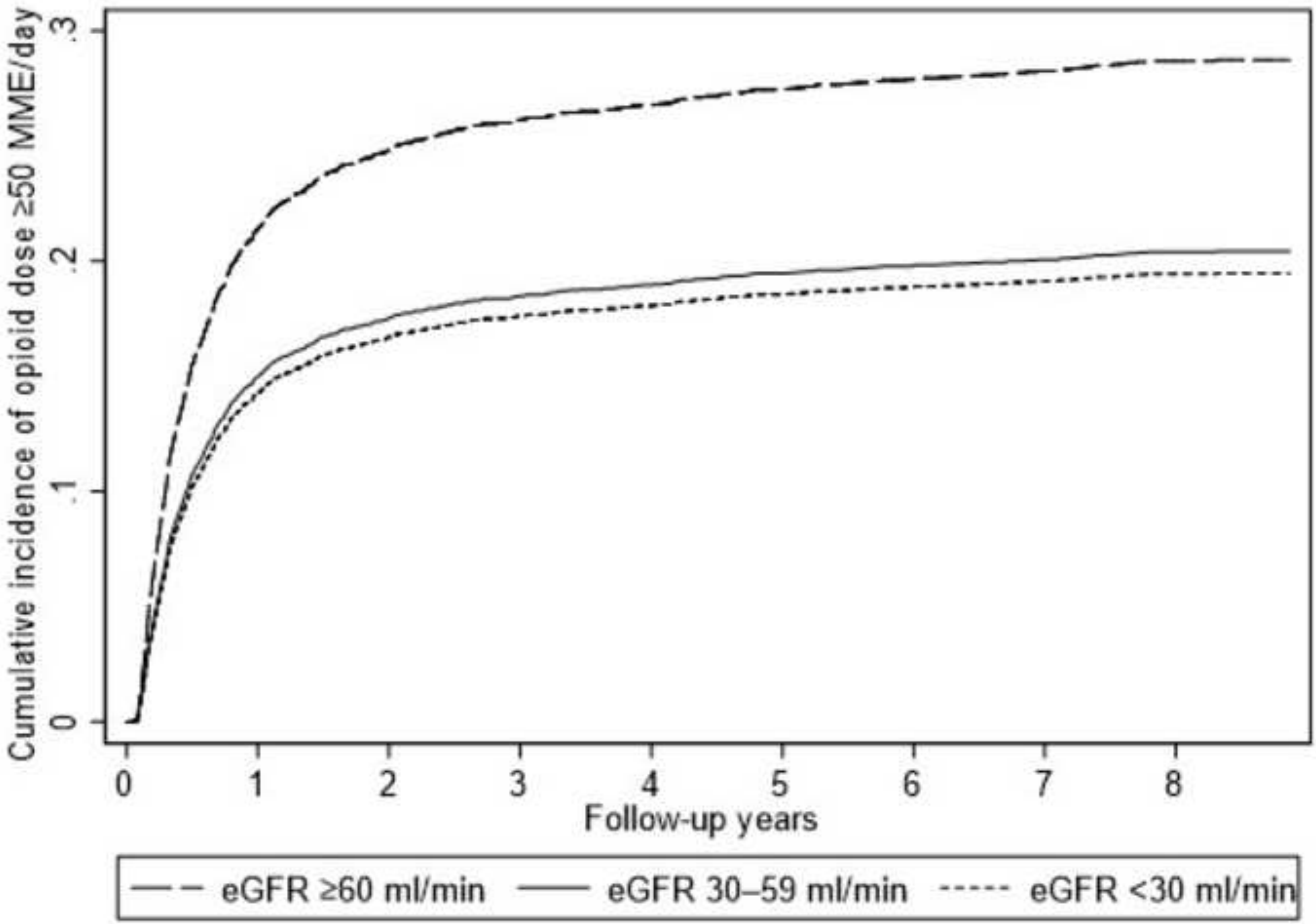

Fig 3

Click here to access/download;Figure;Fig 3.jpg

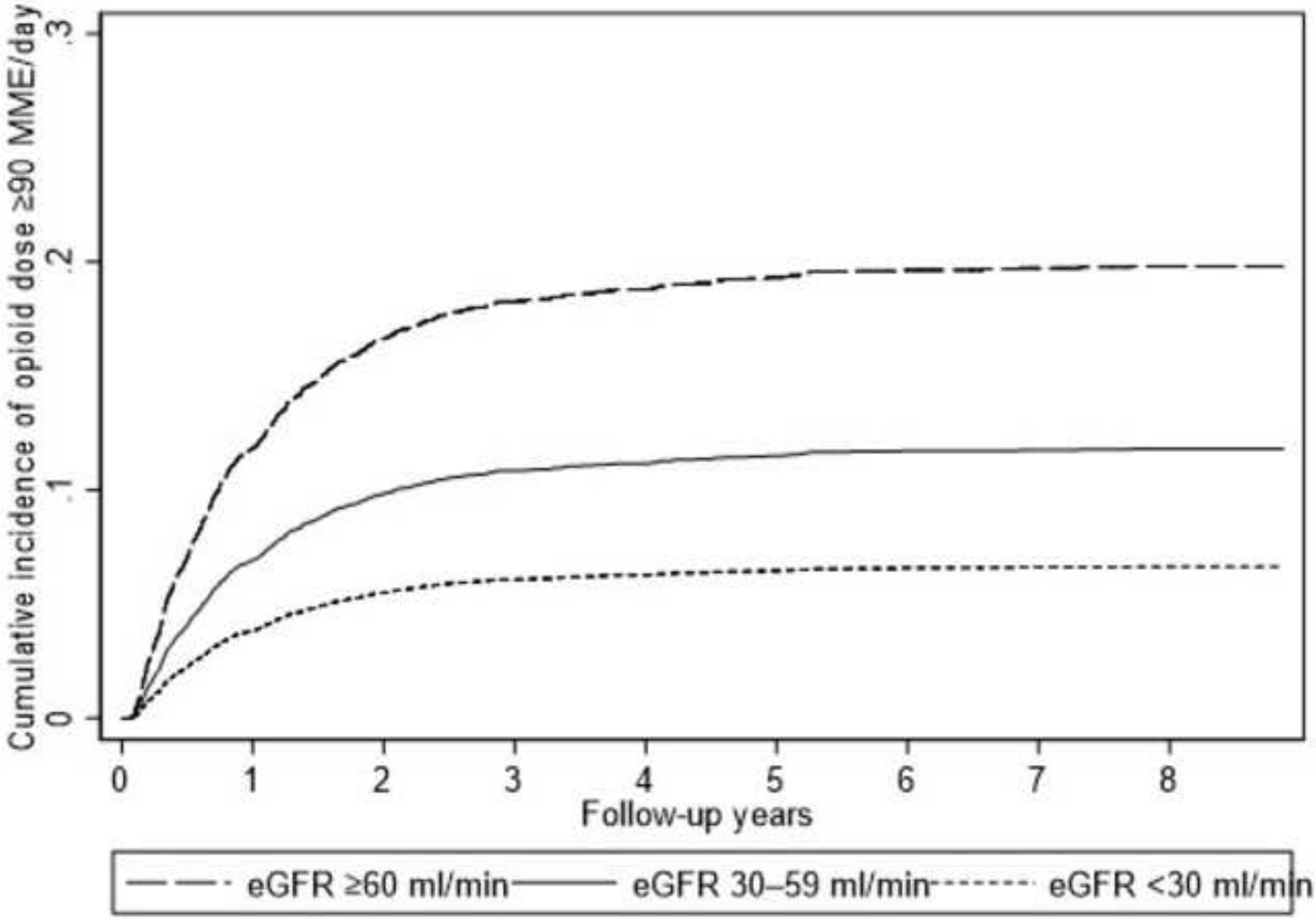

S1 Table

Click here to access/download
Supporting Information
S1 Table.docx

S2 Table

Click here to access/download
Supporting Information
S2 Table.docx

S3 Table

Click here to access/download
Supporting Information
S3 Table.docx

S4 Table

Click here to access/download
Supporting Information
S4 Table.docx

S5 Table

Click here to access/download
Supporting Information
S5 Table.docx

S6 Table

Click here to access/download
Supporting Information
S6 Table.docx

S7 Table

Click here to access/download
Supporting Information
S7 Table.docx

S8 Table

Click here to access/download
Supporting Information
S8 Table.docx

S9 Table

Click here to access/download
Supporting Information
S9 Table.docx

S1 Fig

Click here to access/download
Supporting Information
S1 Fig.docx
