# Supplementary material for: Patterns of opioid dose escalation in patients with chronic kidney disease initiated on opioids for the treatment of non-cancer pain
Source: PLoS One. 2026 Mar 20;21(3):e0345309. doi: 10.1371/journal.pone.0345309 (PMC13004407; doi:10.1371/journal.pone.0345309)
Supplement: Attachment — Submitted filename: Response_to_Reviewers2_PONE-D-25-59036.docx [file pone.0345309.s013.docx]

24 February 2026

Dear Academic Editor

PLOS ONE

**Revision of Manuscript Entitled:** P*atterns of opioid dose escalation in patients with chronic kidney disease initiated on opioids for the treatment of non-cancer pain (*PONE-D-25-59036)

We would like to sincerely thank you for your careful review of our manuscript and for your constructive and insightful comments. We are grateful for the positive evaluation of our work and for the valuable suggestions, which have helped us to improve the clarity, clinical relevance, and overall quality of the manuscript.

We have carefully revised the manuscript in response to all comments. All changes have been highlighted in the revised version using track changes. Below, we provide a point-by-point response to the issues raised.

**Response to Editor’s Comments**

**Comment:**
The manuscript would benefit from further discussion on different types of pain in CKD, non-opioid pharmacological agents, non-pharmacological strategies, and the potential role of dietary phosphorus restriction.

**Response:**
We have expanded the Discussion section to include a more detailed description of pain phenotypes in CKD, alternative pharmacological therapies, non-pharmacological interventions, and nutritional considerations related to CKD–mineral and bone disorders. We have also highlighted the limited empirical evidence regarding dietary phosphorus restriction and framed this as an important direction for future research. These additions are presented in the revised Discussion section (pages 332-348).

**Response to Reviewer #1**

**Comment:**
The article may benefit from discussing different types of pain in CKD, non-opioid pharmacological agents, non-pharmacological strategies, and dietary phosphorus restriction.

**Response:**
Same as above comments (pages 332-348)

**Data Availability Statement**

**Comment:**
Please clarify restrictions on data sharing and provide appropriate access information.

**Response:**
We have revised the Data Availability Statement as below:

*“ The data used in this study originate from Swedish national healthcare registers and are protected under the General Data Protection Regulation (GDPR) and Swedish legislation. Therefore, the data cannot be shared publicly. Researchers interested in accessing Swedish register data may submit an application to the Swedish National Board of Health and Welfare (Socialstyrelsen) in accordance with Swedish law. Information on how to apply for data access is available at:* [*https://www.socialstyrelsen.se/en/statistics-and-data/apply-for-data/*](https://www.socialstyrelsen.se/en/statistics-and-data/apply-for-data/) *or by contacting* [*registerservice@socialstyrelsen.se*](mailto:registerservice@socialstyrelsen.se)*.”*

We believe that these revisions have strengthened the manuscript and addressed all concerns raised by the Editor and reviewers. We sincerely appreciate the time and effort invested in reviewing our work and thank you for the opportunity to revise and improve the manuscript. We hope that the revised version will now be suitable for publication in *PLOS ONE*.

Thank you for your kind consideration.

Yours sincerely,

Che Suraya Zin, PhD
Department of Pharmacy Practice
Kulliyyah of Pharmacy, International Islamic University Malaysia
Email: *chesuraya@iium.edu.my*

Juan-Jesús Carrero, PhD
Department of Medical Epidemiology and Biostatistics
Karolinska Institutet, Stockholm, Sweden
Email: *juan.jesus.carrero@ki.se*
